# Global Mental Health and Nutrition: Moving Toward a Convergent Research Agenda

**DOI:** 10.3389/fpubh.2021.722290

**Published:** 2021-10-08

**Authors:** Thalia M. Sparling, Bryan Cheng, Megan Deeney, Marianne V. Santoso, Erin Pfeiffer, Jillian A. Emerson, Florence Mariamu Amadi, Khadija Mitu, Camila Corvalan, Helen Verdeli, Ricardo Araya, Suneetha Kadiyala

**Affiliations:** ^1^Innovative Methods and Metrics for Agriculture, Nutrition and Health Actions (IMMANA), London School of Hygiene and Tropical Medicine, London, United Kingdom; ^2^Global Mental Health Lab, Teachers College, Columbia University, New York, NY, United States; ^3^Department of Anthropology, Northwestern University, Evanston, IL, United States; ^4^Independent Consultant, Winston-Salem, NC, United States; ^5^Vitamin Angels, Santa Barbara, CA, United States; ^6^Food for the Hungry, Phoenix, AZ, United States; ^7^Department of Anthropology, University of Chittagong, Chittagong, Bangladesh; ^8^Institute of Nutrition and Food Technology, University of Chile, Santiago, Chile; ^9^Health Service and Population Research Department, Institute of Psychiatry, Psychology and Neuroscience, King's College London, London, United Kingdom

**Keywords:** malnutrition, common mental disorders, food security, interdisciplinary, depression, intersectoral research

## Abstract

Both malnutrition and poor mental health are leading sources of global mortality, disease, and disability. The fields of global food security and nutrition (FSN) and mental health have historically been seen as separate fields of research. Each have undergone substantial transformation, especially from clinical, primary care orientations to wider, sociopolitical approaches to achieve Sustainable Development Goals. In recent years, the trajectories of research on mental health and FSN are further evolving into an intersection of evidence. FSN impacts mental health through various pathways such as food insecurity and nutrients important for neurotransmission. Mental health drives FSN outcomes, for example through loss of motivation and caregiving capacities. They are also linked through a complex and interrelated set of determinants. However, the heterogeneity of the evidence base limits inferences about these important dynamics. Furthermore, interdisciplinary projects and programmes are gaining ground in methodology and impact, but further guidance in integration is much needed. An evidence-driven conceptual framework should inform hypothesis testing and programme implementation. The intersection of mental health and FSN can be an opportunity to invest holistically in advancing thinking in both fields.

## Key Messages

- Both malnutrition and poor mental health constitute significant public health burdens globally.
- Each field has undergone conceptual and practical transformations, especially in relation to achieving the Sustainable Development Goals.
- The relationship between mental health and food security and nutrition (FSN) has been increasingly investigated, although usually *ad hoc*, with significant limitations from heterogeneous methods, populations, and sub-types of mental health and FSN.
- Global progress toward health goals will be sooner realized by working toward an empirical framework for hypothesis testing that incorporates common determinants and synergistic dynamics of mental health and FSN.

## Introduction

Malnutrition in all forms is a leading source of disability and disease which affects a considerable proportion of the world's population: 1 in 9 people is hungry and undernourished and 1 in 3 people is overweight or obese (1). Unhealthy diets are among the top three underlying causes of mortality worldwide (2). Moreover, the coexistence of undernutrition and obesity is increasing in several low- and middle-income countries (LMIC), compounding associated health risks (3). Food security, or everyone at all times having access to affordable, safe, sufficient, and nutritious foods (4), is a key determinant of nutritional outcomes such as diet quality, nutrient adequacy, and nutritional status, and thus are considered together here forth.

Another major source of disability and disease is poor mental health. In 2019, mental health was the second leading cause of years lost to disability (YLD) worldwide, accounting for 15% of the total YLD (2). A recent meta-analysis estimated that about 20% of mothers in developing countries experience clinical depression after childbirth (5). LMICs spend on average a mere 0.5% of national health budgets on mental health, despite the fact that they constitute over 80% of the global population (6).

Although they are often thought of as two very separate fields of study, in the context of the internationally agreed Sustainable Development Goals (SDG) on health and well-being (7), there is an increasing recognition that each these areas should be key focal points of action to leave no one behind (8, 9). Furthermore, there is an opportunity to focus on synergies between food security and nutrition (FSN) and mental health. We aim to summarize the developments of both fields in this regard, as well as how they have intersected empirically, and suggest ways forward to advance progress toward global public health goals.

## Development of Global Agendas on Mental Health and Food Security and Nutrition

**Figure 1:**
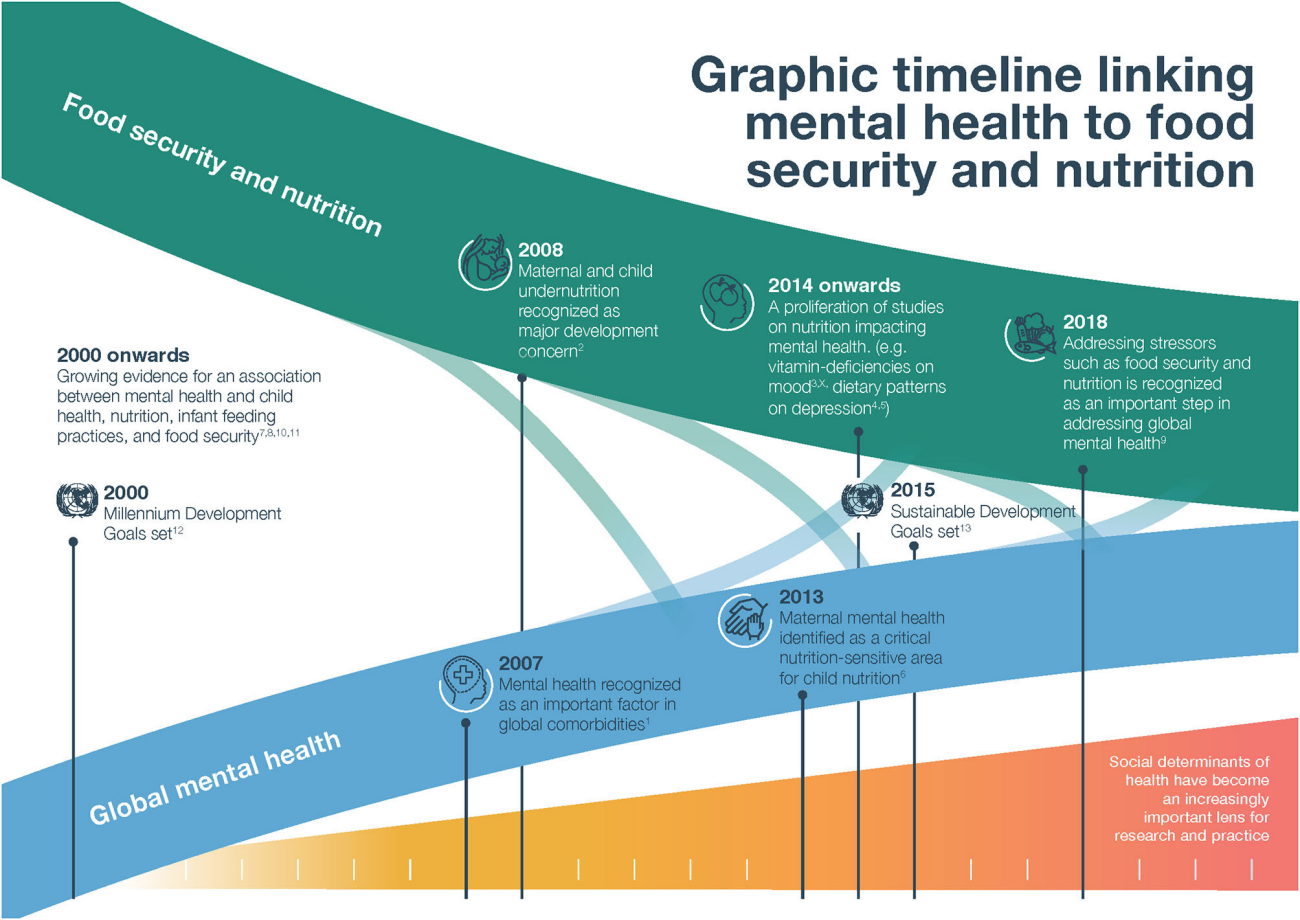
Graphic timeline linking mental health to food security and nutrition.

We trace the progression of both mental health and FSN on the global agenda, marked by the Millennium Development Goals (MDGs) in 2000, to the SDGs in 2015 and beyond (Figure 1). The MDGs focused many of their targets and indicators on health, but linked to nutrition only though a narrow focus on hunger and underweight status (7). Mental health was almost entirely ignored in the MDGs (10). Implicitly, however, both FSN and mental health were recognized by the research community as contributing to and interrelated with the goals of eradicating poverty, promoting gender equality, reducing child mortality, and improving maternal health (11). Since 2007, there has been strident progress toward elucidating these contributions, albeit as largely separate fields.

Prince et al. contributed a paper on the state of evidence on global mental health in 2007, arguing that achieving global health goals would not be possible without addressing mental health (12). It described mental health as both a precursor to and an outcome of other major health burdens, such as parental depression compromising child care or diabetes and obesity fueling poor mental health. A Lancet series in 2008 focused on maternal and child undernutrition similarly made the case that investing in nutrition actions (specifically the impact of scaling 10 effective nutrition-specific interventions to 90% in 36 high-burden countries), would be a significant stride toward achieving global health goals (13).

Both fields underwent transformations as the MDG period came to its conclusion, situating respective burdens within a broader context. Prevailing evidence coalesced around nutrition-sensitive interventions and enabling environments (13). For FSN, this evidence signaled the importance of underlying factors such as agriculture, food environments, caregiving and gender dynamics, and poverty in limiting the success of even high-quality, large scale nutrition-specific interventions (14). For mental health, this wider perspective was more nascent, though it was apparent in the growing body of evidence showing the potential positive effects of addressing maternal mental health, especially for children's health (15).

Within the UN's Development Agenda for the post-2015 era, the 17 Sustainable Development Goals (SDGs) incorporated a broader view of FSN. SDG 2 to “End hunger, achieve food security and improved nutrition and promote sustainable agriculture” included elements of agricultural productivity, diversity, and sustainability. The Global Nutrition report in 2017 made a clear case for the importance of improving nutrition in achieving at least five SDG goals (16). However, FSN targets and indicators are still missing important aspects of global food systems and environments.

The focus on mental health lags behind FSN within the SDGs. Goal 3 brought non-communicable diseases into focus, with target 3.4: “By 2030, reduce by one third premature mortality from non-communicable diseases through prevention and treatment and promote mental health and well-being” (17). However, mental health as a part of NCDs is rarely mentioned, and the official indicator is suicide prevention, hardly reflecting the breadth of impact of mental health problems. A more comprehensive commitment was made via the 3rd High-Level Meeting of the United Nations General Assembly on the Prevention and Control of NCDs, especially in point 11 of the political declaration which states: “depression alone affects 300 million people globally and is the leading cause of disability worldwide” (18).

From 2018, there was an extensive re-framing of both nutrition and global mental health related to sustainable development. The discourse in each discipline shifted further from etic, clinical health arguments to social determinant frameworks that embedded mental health and FSN across the spectrum of environmental, (psycho)social, and biological factors. Researchers and practitioners have explicitly tried to situate mental health against and within almost every one of the 17 SDG goals (8), although it remained persistently underfunded despite the magnitude of the burden. Nutritional problems (including the double burden of nutrition-related chronic conditions and undernutrition) were presented through a broader political and societal lens, including climate change and environmental threats (3, 19).

## State of Current Knowledge and Synergies in Research

The trajectories of research on mental health and FSN are further evolving into an intersection of evidence. Common mental health disorders, depression, stress, and anxiety, have been associated with increased food insecurity in many contexts (20). Specific micronutrients and dietary patterns have also been investigated as they related to mental health across settings and populations with varying results (21–25). Studies have shown associations between poor mental health and poor diets, low intake of certain nutrients, and low and high Body Mass Index (26, 27). However, not all evidence supports these associations between mental health and FSN (28, 29).

As a determinant, mental health has been identified as an important driver within the complex theoretical pathways connecting agriculture, food systems, nutrition, and nutrition-related health outcomes. Much of this work has focused on maternal and child health (30, 31). Depression and stress, which are often characterized by low motivation and drive, poor appetite, neglect of oneself and others, were linked early on to sub-optimal breastfeeding practices and preterm birth (32, 33). Poor mental health of parents, especially mothers, has been associated with poor child growth outcomes and illness across contexts (34), but these findings are not consistent (28). Efforts to understand and prevent obesity have drawn attention to the importance of mental health and feeding behaviors (35), although the evidence remains equivocal (36).

Mental health and FSN are also linked through a complex and interrelated set of determinants, including diverse factors of poverty, physiology, emotional stress, conflict, harmful gender norms, domestic violence, and lack of control over economic resources (37–39). Moreover, vulnerable groups such as the elderly, low-income adults, pregnant and lactating women and children, among others, tend to accumulate disproportionate burdens of both poor mental health and poor nutrition outcomes as a product of inequity (1, 40).

Some links are still unexplored or just emerging. Many investigations into mental health are not sensitive to food and nutrition aspects, and vice versa. Other aspects, such as the mental health benefits of food production, empowerment, and the impacts of food environments on mental health have only begun to be explored in a more systematic way. For instance, gardening programs have offered benefits beyond food provision for caregivers of those with dementia in Uganda (41). Other evidence may be supported by similar causal mechanisms, providing a stronger theoretical framework for the links between the two. It is likely that the Covid-19 pandemic will bring to light further evidence on the links between mental health and nutrition (42).

## Discussion: Current Gaps and Challenges

We explore next the current challenges in understanding how mental health is related to FSN, and what might be promising approaches to address these interconnected burdens.

### Hypothesis Testing and Causal Inference

There is now a plethora of correlational studies linking mental health and FSN using various measures in diverse populations. However, much of this evidence, even if the research hypothesis is built in one direction, does not unpack causal mechanisms. For instance, food insecurity is known to cause increased stress and worry, which could manifest as depressive symptoms (20). Other research shows that women who are depressed often express less motivation or less interest in self-care and bonding with others, which in turn could mean eating a less healthy diet, less care in preparing meals, and less attention to children's food intake (43). Mental health could therefore be hypothesized as an outcome of food and nutrition insecurity, or an exposure preceding it.

In another example, it is almost universally assumed that maternal depression causes worse child feeding practices, less caretaking, and poor child growth, and is discussed as such even in cross-sectional studies (34, 44). But it is quite possible that poor child outcomes are cause for poor mental health, especially where child health is seen as a reflection of the mother and the family (45). There are biologically plausible and proven ways in which a lack of key nutrients can impede optimal neurotransmission and depress moods, but also ways in which poor mental health causes appetite and dietary changes, thus potentially impacting nutritional status.

The heterogeneity of the evidence base—including differences in theoretical and analytical approaches, intervention design, screening tools and measures, validation of tools, timing of measurement, and populations of interest—limits the inferences we can make overall about these important dynamics. Even in the case of systemic interactions between FSN and mental health that result in feedback loops, an evidence-driven conceptual framework should inform hypothesis testing in studies. As it stands, the relationship between mental health and FSN is often investigated *post-hoc* or as secondary analyses.

### Measurement Approaches

Many researchers across mental health and FSN are designing interdisciplinary projects that draw on one another, and in the process are grappling with how to integrate methods and measurements from outside their core expertise. There is increasing demand from both groups of researchers for further theoretical and technical guidance.

There is now substantial measurement guidance for food security, nutrition, and nutrition-sensitive outcomes (46, 47), from the most direct indicators for nutritional status such as anthropometry and micronutrient deficiencies, to proximal or underlying factors such as diet quality and food security or poverty. Although there are considerable contextual and methodological limitations, there is a biophysical element to nutrition that is common to all people and thus somewhat more objectively measured. The array of these measures has been the focus of intensive development and validation efforts in the last decades, especially those designed for LMIC and/or fragile contexts.

There is also a developed discourse on measuring common mental disorders in LMIC, and summary guidance of tools to do so (48). However, measuring mental health with standard tools across contexts is particularly challenging. These challenges stem from cultural differences in epistemology, cross-contextual equivalence, the vast, deep and diverse issues of stigma around poor mental health, translocation, and differences in training and implementation of mental health services (49, 50). Especially for mental health, many measures rely on nosological distinctions and clinical assessment, which may be too narrow to capture myriad intersectoral and interdisciplinary outcomes that we now consider important. That said, the experiences of mental health problems around the world have been shown to have commonalities (51). New approaches are underway to signal both common elements and the contextual nuances of mental health in a population, such as moving away from disease classification and focusing more holistically on symptoms (52–54).

Screening tools are the most common approach to determine population prevalence of common mental disorders in low-resource settings. *In situ*, there are often weaknesses in the validation process, proper adaptation and translation, both cultural and technical, training for those asking these modules, the construction of analyses, and the interpretation of those results. Clinical interviews, the common gold standard in mental health, may not themselves be validated for certain contexts, especially LMIC (51). If screening tools are validated against clinical interviews, this may introduce even greater bias. Several approaches have been established to overcome these issues, including using concurrent validity, defining the gold standard locally, or establishing new types of screening tools (55–57).

Connecting mental health and FSN presents its own methodological challenges. For instance, dietary assessment is predominantly based on recall and perception, and therefore could be biased by mental health status as mood deeply influences perception. However, the association between the two is in many ways intrinsic. Not having access to affordable, healthy food exhibits as increasing anxiety, worry, and depression in high-income (58, 59) and LMICs alike (60). Improving both burdens will require deeper understanding of how mental health conditions are experienced and related to FSN in various populations.

### Interventions and Programmes

Where the MDGs did include FSN and mental health, the focus was to integrate assessment and services into primary care, and as such most programmes were clinically focused. Integration and multi-sectoral approaches to both FSN and mental health then expanded laterally to the continuum of care, from community-based initiatives to acute and emergency humanitarian response.

Low cost mental health interventions, involving non-specialized or community health providers, have begun to emerge and often show positive impacts for individuals of all ages, including caregivers and children (61, 62). FSN programs working to improve gender disadvantage, social cohesion, peer support, decision-making, and agricultural practices may also act on mental health status, which in turn may strengthen engagement with food and nutrition behaviors (63).

Along those lines, some current FSN projects with no direct mental health intervention components are beginning to assess impact on mental health outcomes based on the hypothesis that improvements in FSN will in turn improve mental health. The findings of these interventions are still emerging. Both mental health and FSN outcomes have been treated as a de facto vulnerability metrics. For instance, measures of self-efficacy, depressive symptoms, or anxiety have been used to measure latent characteristics of resilience and well-being (64). Nutrition outcomes are often used as proxy measures of resilience (65). Although there are examples of studies including both mental health and FSN components, collectively they have not necessarily been part of strategic planning and the results have not been systematized.

There are new opportunities for connecting mental health and FSN research, although indeed the nutrition field is further along in their integration efforts. Given that both mental health and FSN have complex determinants, research agendas that include both will have to carefully consider their approach. For instance, water, sanitation and hygiene, care practices, social and gender dynamics, violence and conflict, and poverty have been independently connected to both FSN and mental health. Ever-advancing analysis methods will serve this agenda. There are risks of increasing the complexity of research and programs, potentially burdening implementation and research staff and losing focus by trying to incorporate too many goals. Even if this is warranted in local contexts, there may be trade-offs in building models that are appropriate to scale. Another risk is presenting analyses that are theoretically ungrounded.

## Future Directions

While both fields have independently incorporated social determinant perspectives, there is an opportunity to strengthen understanding and action on how to leverage both to improve both FSN and mental health outcomes. The intersection of mental health and FSN can be an opportunity to invest holistically in progress toward the SDGs while advancing the thinking on dynamics between and within mental health and FSN.

In 2020, the Agriculture, Nutrition and Health (ANH) Academy constituted a working group of interdisciplinary experts on mental health and food and nutrition, as well as methodologists with expertise in the design and evaluation of implementation research programmes in these fields. The aim of this group is to synthesize what is known about the intersection of these themes and promote more systematic thinking and action in research, programs, and policies. Its three objectives are: (1) assess the current state of knowledge on mental health as it relates to FSN; (2) prioritize key gaps and questions that need to be filled or answered in order to effectively aid research in this nexus; and (3) develop guidance and resources on best practice for applied research on linkages between mental health and FSN.

The Covid-19 pandemic has shown how fragile progress has been in both FSN and mental health. As a result of the social and economic upheaval associated with the pandemic, combined with the decrease of health care access and resources, FSN and mental health burdens are already rising, and it is estimated that they will continue to increase in the next years. Even so, the pandemic may elucidate important connections between FSN and mental health and provide an opportunity to learn about these intersections.

Thus, it is even more timely and urgent to make progress in these areas. Systematic thinking in this space will move the research community toward frameworks of investigation and action on these important issues. In the post-Covid-19 era, progress toward global FSN and better mental health are important to achieving the most universal goals of health and well-being for all.

## Author Contributions

TS conceptualized the work with BC, supported by the entire ANH Academy Working Group on Mental Health. BC, MD, MS, EP, JE, FMA, KM, CC, HV, RA, and SK each provided feedback and revision throughout the drafting process. TS reconciled all contributions and prepared the final manuscript, which was approved by all authors.

## Funding

This work was supported by the Innovative Methods and Metrics for Agriculture, Nutrition and Health Actions (IMMANA) Programme, funded by UK Foreign Commonwealth and Development Office (FCDO), grant number 300654, and the Bill and Melinda Gates Foundation, grant number INV-002962.

## Conflict of Interest

The authors declare that the research was conducted in the absence of any commercial or financial relationships that could be construed as a potential conflict of interest.

## Publisher's Note

All claims expressed in this article are solely those of the authors and do not necessarily represent those of their affiliated organizations, or those of the publisher, the editors and the reviewers. Any product that may be evaluated in this article, or claim that may be made by its manufacturer, is not guaranteed or endorsed by the publisher.
